# The Amyand's Hernia: A Rare Clinical Entity Diagnosed by Computed Tomography

**DOI:** 10.1155/2013/638270

**Published:** 2013-04-10

**Authors:** Suat Keskin, Cihan Şimşek, Zeynep Keskin

**Affiliations:** ^1^Department of Radiology, Meram School of Medicine, Necmettin Erbakan University, P.O. Box 42080, Konya, Turkey; ^2^Department of Radiology, Konya Training and Research Hospital, Konya, Turkey

## Abstract

Amyand's hernia, named for the first person to describe an inguinal hernia containing the vermiform appendix, is an uncommon variant of an inguinal hernia. Amyand's hernia is an extremely rare condition and is often misdiagnosed. Traditionally, these hernias have been diagnosed at surgery but are increasingly diagnosed by abdominal computed tomography (CT) scans. CT of the abdomen may help in guiding the diagnosis.

## 1. Introduction

The finding of vermiform appendix in inguinal hernia is called Amyand's hernia. Appendix within inguinal hernias is uncommon, with a reported incidence varying from 0.28% to 1% [1, 2]. Traditionally, these hernias are diagnosed at surgery but now diagnosed by abdominal computed tomography scans. We present a rare case of inguinal hernia with presence of appendix in a 51-year-old man.

## 2. Case Report

**Figure 1:**
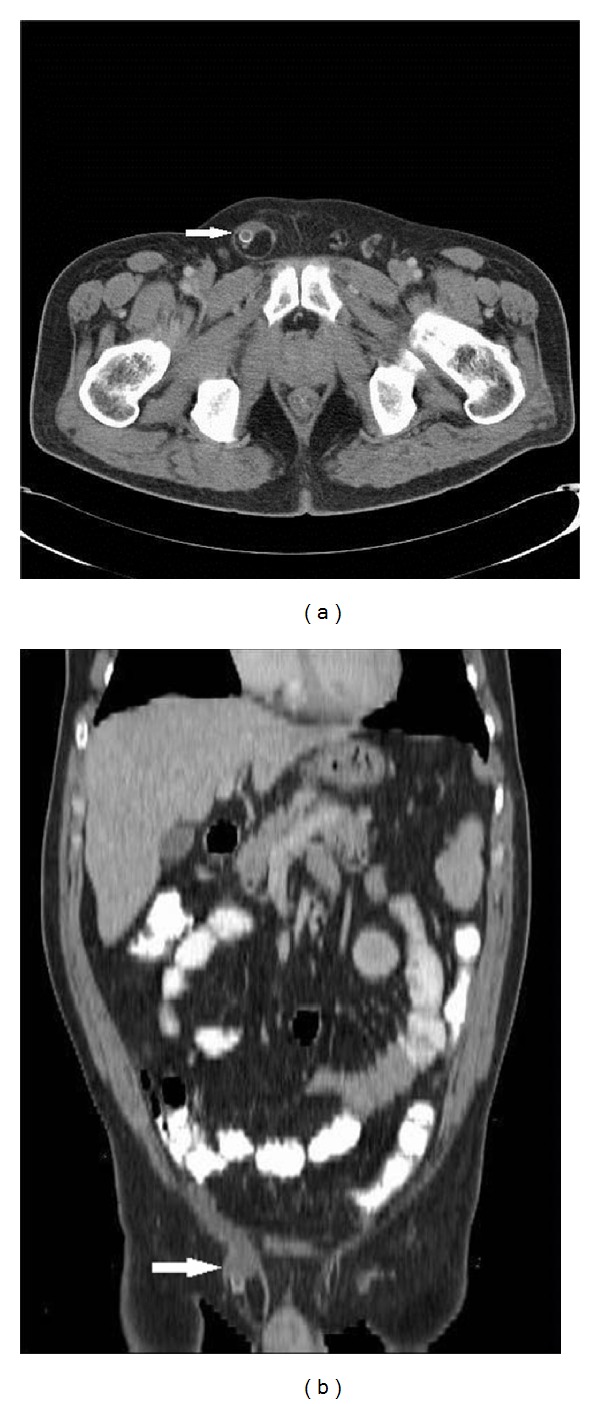
Amyand's hernia was showed after intravenous (IV) contrast CT of the abdomen. Axial (a) and coronal MPR (b) images. A small amount of fluid around appendix with appendiceal fecalith is seen on the axial image (arrow). The inflamed appendix is particularly well, as seen on the coronal reformats (arrow).

A 51-year-old man was admitted to our institution with one-day history of right inguinal pain and swelling. A walnut-sized mass was found, located lower than the inguinal ligament. Severe pain was induced by direct compression. Physical findings revealed inguinal tenderness and rebound mainly in the right lower quadrant. There were no complaints of nausea or vomiting. In this case of a suspected incarcerated hernia the hernia could not be reduced. The patient had increased white blood cell count (12 300/*μ*L, with 82% neutrophils). CT revealed an appendix from pericecal area extended to the right inguinal channel. A small amount of fluid was present around, also Figures 1(a) and 1(b) with an appendiceal fecalith (see also Figures 1(a) and 1(b)). The wall of appendix was thickened. At surgery, an inflamed appendix with appendiceal fecalith was found within the hernia sac. The appendix was removed and hernia repair was performed.

## 3. Discussion

There are several clinical manifestations of Amyand's hernias: reducible or incarcerated hernia with noninflamed appendix or inflamed appendix (hernia appendicitis). The presentations of vermiform appendix within inguinal hernia sac are called Amyand's hernia [3, 4]. Ultrasound and/or CT scan can make a correct diagnosis [5, 6]. The presence of an inguinal hernia is usually obvious on CT scans, particularly in males. To confirm the presence of an Amyand's hernia, the sagittal and coronal reformats are particularly useful in identifying the blind-ending tubular appendix arising from the caecum and entering the inguinal canal. The size of the appendix is unreliable for diagnosis as collapsed small bowel loops can be similar in caliber. Radiologists need to be aware of the Amyand's hernia. It is important to recognize both the inflamed and the normal appendix within the inguinal canal, as well as abdominal complications.

## 4. Conclusion

Amyand's hernia is an extremely rare condition and is often misdiagnosed. CT of the abdomen may help in guiding the diagnosis as in our case.
